# Avian Leucosis Virus-Host Interaction: The Involvement of Host Factors in Viral Replication

**DOI:** 10.3389/fimmu.2022.907287

**Published:** 2022-05-26

**Authors:** Shuang Tang, Jie Li, Yung-Fu Chang, Wencheng Lin

**Affiliations:** ^1^ Guangdong Provincial Key Laboratory of Agro-Animal Genomics and Molecular Breeding, and Key Laboratory of Chicken Genetics, Breeding and Reproduction of Ministry of Agriculture, College of Animal Science, South China Agricultural University, Guangzhou, China; ^2^ Department of Population Medicine and Diagnostic Sciences, College of Veterinary Medicine, Cornell University, Ithaca, NY, United States

**Keywords:** avian leukemia virus, interaction, immunosuppression, host factors, replication

## Abstract

Avian leukosis virus (ALV) causes various diseases associated with tumor formation and decreased fertility. Moreover, ALV induces severe immunosuppression, increasing susceptibility to other microbial infections and the risk of failure in subsequent vaccination against other diseases. There is growing evidence showing the interaction between ALV and the host. In this review, we will survey the present knowledge of the involvement of host factors in the important molecular events during ALV infection and discuss the futuristic perspectives from this angle.

## Introduction

Avian leukemia virus (ALV) is an oncogenic retrovirus associated with tumorigenic disease, decreased fertility and growth retardation (1–3). Moreover, this causative agent causes severe immunosuppression, increasing susceptibility to other microbial infections and the risk of failure in subsequent vaccination against other diseases (4). Viral transmission of this disease mainly occurs through a vertical route from the hen to offspring *via* infected embryos (5). However, it can also be transmitted horizontally, following direct and indirect contact with infected chickens or virally contaminated fomites (6). Untill now, no effective vaccines and drugs are available to prevent or control this disease. Therefore, clinically, the most effective way to control this disease is the differentiation and eradication of ALV-infected individuals in the population (7).

ALV belongs to the *Alpharetrovirus* genus of the family *Retroviridae*, which is composed of a single positive-stranded RNA dimer. Viral genome is approximately 7.8 kb in length and contains three main coding genes: *gag* (encoding the internal structural proteins of the virion), *pol* (encoding RNA-dependent DNA polymerase) and *env* (encoding the envelope glycoprotein). Based on the viral cross-neutralization patterns, host range and viral envelope interference, ALV are usually classified into 11 subgroups, including subgroups A, B, C, D, E, F, G, H, I, J and K. The members of subgroups A, B, C, D, J, and K are exogenous viruses, which were found in naturally infected flocks (8).

As a retrovirus, the replication cycle of ALV contains six steps: binding and entry, uncoating, reverse transcription, provirus integration, virus protein synthesis and assembly, and budding. Based on the applications of protein-protein, protein-RNA or protein-DNA interaction detection methods, the ALV-host interactions in different molecular events are found at protein and RNA levels. The interaction between ALV and host factors (including protein, miRNA, and LncRNA) leading to the pivotal steps of the viral life cycle are critical implications for understanding the pathogenesis and developing novel strategies to prevent or control this disease. This review mainly focuses on the recent findings of the interactions of ALV with host factors, surveying the present knowledge of the involvement of host factors in the important molecular events during ALV infection, and discussing the future perspectives in this angle.

## Cellular Receptors Associated With Viral Entry

As an enveloped virus, ALV directly utilizes membrane fusion to initiate the entry into target cells. The fusion process, followed by importing sub-viral particles into the target cells, is initiated by binding viral envelope glycoprotein to the specific cellular receptors (9). Although the underlying mechanism regarding the entry pathway of ALV is still unclear, several host proteins that serve as cellular receptors on the cellular surface have been confirmed to associate with viral entry. The subgroup specificity of ALV has been mapped to the surface glycoprotein (SU) domain of the envelope (Env) glycoprotein which is responsible for receptor binding (9). Each viral subgroup is highly specific as to the envelope glycoproteins and receptor usage. Based on the binding specificity of the viral envelope, cellular receptors can be roughly classified into four categories: Tva, Tvb, Tvc, and Tvj (10). Tva protein is the receptor shared by the ALV-A and ALV-K; Tvb protein is the receptor shared by ALV-B, ALV-D and ALV-E. For Tva and Tvb, multiple variants have been identified due to the frame-shift deletions and amino acid substitutions. Tvc is the receptor for ALV-C, which is associated with the member of the immunoglobulin protein Ig family (11). Tvj protein contains two members: Na^+^/H^+^ exchanger type 1 (chNHE1) and chicken annexin A2 (chANXA2).

Tva protein is the receptor shared by the ALV-A and ALV-K, possessing high homology with the ligand-binding region of the human low-density lipoprotein receptors (LDLR) (12, 13). The complexity and specificity of this binding drive viral evolution to alter their envelope glycoprotein sequence, making more proteins on the cellular surface act as receptors. The receptor-defective alleles *tva^r1^* (substitution C40W), *tva^r2^* (frame-shifting four-nucleotide insertion), *tva^r3^*, *tva^r4^* (deletion within the first tva intron), *tva^r5^* and *tva^r6^* (deletion within the first tva intron) increase the genetic resistance to ALV-A (14–16), but does not compromise production performance. Interestingly, a series of amino acid residues involving ALV-A Env binding was identified within the single cysteine-rich domain delineated between residues C11 and C50. The amino acid residue Cys38 plays a critical role in Tva binding to ALV-A SU. The formation of a reactive thiolate at Cys38 (Cys38-S-) was induced when Tva binds to ALV-A SU. If the chemical and genetic inactivation of Cys38-S- occurred, the fusion and infection of ALV-A completely failed. However, the Cys38-S- does not involve the SU-TM disulfide bond’s isomerization and Tva-induced TM’s activation (17). A recent report indicated that ectopic expression of chicken *tva* gene in mammalian cells confers susceptibility to ALV-A and ALV-K, In contrast, the knockdown of *tva* gene repairs both viruses’ susceptibility in chicken DF-1 cells (12). Additionally, the amino acid residues G196 and R198 located in HR1 region of the glycoprotein of ALV-K, have been confirmed to associate with the viral entry (18). These data provide evidence for the same receptor shared by ALV-A and ALV-K.

Tvb protein is the receptor shared by ALV-B, ALV-D, and ALV-E, belonging to the tumor necrosis factor receptor family (19). A series of Tvb variants have been identified, including *tvb* ^s1^, *tvb* ^s3^, *tvb* ^t^, *tvb^r^*, *tvb^r2^*, *tvb^r3^*, *tvb^r4^* and *tvb^r5^* (20, 21). Tvb^S1^ confers susceptibility to ALV-B, ALV-D and ALV-E. A single amino acid change (C62S) in the cysteine-rich domain (CRD) of Tvb^S1^ produces the variant Tvb^S3^, causing the loss of binding ability to ALV-E SU (22). Tvb^T^ was identified in the turkey, conferring susceptibility to ALV-E (21). Tvb^r^ containing an in-frame stop codon was found in the inbred chicken line 7_2_, resulting in the loss of viral entry of ALV-B, ALV-D and ALV-E (23). Tvb^R2^ with C125S substitution in CRD3 could effectively reduce the susceptibility to ALV-B and ALV-D infection and nearly eliminates ALV-E infection (24). Tvb^R3^ with C298T substitution significantly reduces the binding affinity to SU, causing the loss of the susceptibility to ALV-B, ALV-D and ALV-E (25). Recently, two Tvb variants designated Tvb^R4^ and Tvb^R5^ were identified, which had insert “AG” between amino acid residues 291 and 292, and “A” between amino acid residues 359 and 360, respectively. Both insertions induced the generation of truncated Tvb proteins, causing the partially functional loss of receptors for ALV-B, ALV-D, and ALV-E (26). Several amino acid residues involving the binding and viral entry were identified in the Tvb. For ALV-B and ALV-D, the amino acid residues 32 to 46 in CRD1 of Tvb^S1^ are sufficient for the function as the receptor, and the amino acid residues Leu-36, Gln-37 and Tyr-42 are critical for the functionality of the receptor (27). For ALV-E, amino acid residues Tyr-67, Asn-72 and Asp-73 in CRD2 of Tvb^S1^ are essential for viral binding and entry (28).

The first identified receptor of ALV-J is the multi-spanning transmembrane protein chNHE1, containing 12 TM domains, 6 extracellular loops (ECLs), and a long intracellular C-terminal tail (29–31). The first ECL (ECL1) is critical for the function of chNHE1 by directly interacting with ALV-J SU. The amino acid residues 28 to 39 of the N-terminal membrane-proximal region of ECL1 are the minimal domain for chNHE1 binding to SU. Especially, the residues A30, V33, W38 and E39 are critical for the binding ability of chNHE1 (24, 32). Deletion or substitution of W38 of chNHE1 could abrogate its binding to ALV-J SU (33). Interestingly, the amino acid residues 38 to 131 of the N terminus and 159 to 283 of the C terminus of ALV-J SU are critical for its binding ability to chNHE1 and viral infection (34). Additionally, several glycosylation sites in SU involved its binding ability to chNHE1, including N6, N11, N17 and N193. The glycosylation sites N6 and N11 play a crucial role in receptor binding and viral entry (34, 35). The glycosylation site N193 plays a critical role in viral replication; mutating N193 weakens its binding ability to chNHE1 (35).

## Host Immune Response Against ALV

### Involvement of Host Innate Immune Response During ALV Infection

Innate immunity provides the first defense line against the invasion of pathogenic microorganisms (36). However, ALV has been confirmed to suppress the host’s innate immune response (37, 38). Studies on the suppression mainly focused on the effects of ALV on immune cells, such as monocyte, dendritic cells (DCs), and macrophages.

Monocyte are the precursor cells of macrophages and DC cells, which plays an important role in innate and adaptive immunity (39). Monocyte infected with either field ALV-J strain or laboratory strains cannot differentiate from macrophages because of cell death induced by ALV-J. This inhibition is associated with the up-regulation of interleukin 1β (IL-1β) and IL-18 and the increased activities of caspase-1 and caspase-3 (40). Although ALV-J infection-induced monocyte death, the underlying mechanism is still unclear. More effort will be required to study the effects of ALV on monocyte differentiation.

Macrophage is well known for their pivotal roles in innate immunity. If macrophages are activated by viruses, microbes or cytokines, it usually plays a critical role in pathogen clearance, immunomodulatory, and tissue integrity maintenance through secreting pro-inflammatory cytokines (41). It has been reported that, compared to the mock-infected control, the monocyte-derived macrophages (MDM) infected with ALV-J strain SCAU-HN06 secrets more interferon β (IFN-β) and IL-6, and less IL-10 (42). Similarly, Long-chain acyl-CoA synthase-1 (ACSL1), a member of the ACSLS family was identified as interferon-stimulated genes (ISG), induces inflammatory response through the PI3K/Akt signaling pathway in MDM, then inhibits ALV-J replication through type I IFN signaling (43, 44). These data provided clues that ALV-J may induce host innate immune response through activating MDM. In contrast, our previous study indicated that ALV-J possesses an inhibitory effect on type I interferon production in chicken macrophages HD11 cells. When host cells were infected with ALV-J, the IκBα phosphorylation is blocked, causing the IκBα accumulation in the cytoplasm. The accumulated IκBα make NF-κB/IκB complex stabilized, prevents the NF-κB from transferring into the nucleus, and finally suppresses the interferon expression (37). However, how does ALV-J inhibit the phosphorylation of IκB and which component (viral protein, nucleotide or something else) of ALV-J affect type I interferon expression and the activation of the NF-κB are unknown. Interestingly, in the macrophage RAW264.7 stably expressing the p27-GFP fusion protein, the expression of TNF-α, IL-1β, IL-6 and IL-12, and the proliferative activity stimulated by LPS were specifically suppressed, providing evidence for the association of P27 with ALV-induced immunosuppression (38). More effort will be required to reveal the effect of ALV-J on the innate response systematically.

DCs are the sentinel cells of the immune systems (45), playing critical roles in pathogen recognition, antigen presentation and T cells stimulation (46). In ALV-J infected bone marrow-derived DCs (BM-DCs), cellular differentiation and maturation were effectively inhibited, following the occurrence of apoptosis *via* the aberrant expression of microRNAs (47). Interestingly, in the surviving BM-DCs infected with ALV-J, the expression of Toll-like receptor 1 (TLR1), TLR2, TLR3, Major Histocompatibility Complex I (MHC I), MHC II and pro-inflammatory cytokines were significantly decreased (48). The inhibition of differentiation and maturation and the occurrence of apoptosis of DCs may involve the immunosuppression of the host innate immune response during ALV-J infection.

### Association of Host Adaptive Immune Response With ALV Infection

Previous reports indicated that a strong immune response was present in spleen of ALV-J infected chicken at 2 weeks of age, and the immune response rapidly decreased at 4 weeks of age. This finding provides evidence that 3~4 weeks post-infection may be the critical period for ALV-J inducing immunosuppression (49). CD8^+^ T cell response is vital in host adaptive immune response. CD8^+^ T cell response triggered by ALV-J was obviously observed at 7 dpi in peripheral blood lymphocytes (PBL), antibodies against ALV-J can be detected at 21 dpi and then increased slightly. However, the decrease in the ratio of CD4^+^/CD8^+^ could be observed in the thymus at 14 dpi and in the PBL at 21 dpi, implying the period of immunosuppressive effect induced by ALV-J (50). A recent report confirmed that CD8^high^ αα^+^ T cells represent an effective response to viral infection, but CD4^+^CD8^low+^ T cells involve the negatively regulate the activity of T cells (51). ALV-J infection caused severe immunological tolerance, showing the absence of specific antibodies against the virus. If chicken infected with ALV-J, the bursa of Fabricius were poorly developed and the bursa follicles cannot differentiate into cortex and medulla due to the blocking of the differentiation of CD117^+^chB6^+^ B cell progenitors, causing development arrest of B cells and the inhibition of humoral immunity (52). Additionally, tyrosine kinase Lyn (a key protein in the BCR signaling pathway) involved B cell anergy by inhibiting BCR signal transduction (53). Briefly, ALV-J SU interacts with Lyn, induces phosphorylation of Lyn at amino acid residue 507, activates the negative regulatory effect of Lyn on the BCR signal transduction pathway, and then mediates B cell anergy (53). Regulatory T cells (Tregs) are a subset of mature T cells with negative immunomodulatory effects (54). a recent study reported that Tregs play a moderately important role in ALV-J infection. Upon ALV-J infection, the amount of CD4^+^CD25^+^ Tregs was increased in the blood and immune organs significantly and suppressed the proliferation and activation of B cells through expression of TGF-β and CTLA-4 (55).

## Cellular Factors Affecting ALV Replication

### Non-Coding RNA Involved ALV Replication

The non-coding RNAs (ncRNA) contain circle RNA (circRNA), micro RNAs (miRNA) and long non-coding RNA (lncRNA), performing certain biological functions (56). Several miRNAs have been confirmed to affect ALV replication. During ALV-J infection, the expression of miR-23b was up-regulated, and the expression of its target gene interferon regulatory factor 1 (IRF1) was increased. The miR-23b could promote ALV-J replication by targeting IRF1 *via* affecting the type I IFN signaling pathway (57). Similarly, miR-34b-5p promotes ALV-J replication by inhibiting the melanoma differentiation associated gene 5 (MDA5) signaling pathway (58). Gga-miR-200b-3p acts as a facilitator of ALV-J replication by targeting the host protein dual-specificity phosphatase 1 (DUSP1) (59). In contrast, gga-miR-1650 inhibits ALV-J replication through binding to the 5’UTR of the viral genome (60). Gga-miR-221 deregulates the G1/S transition by targeting cyclin-dependent kinase inhibitor 1B (CDKN1B), promoting cell proliferation and cell cycle progression (61). Gga-miR-375 and gga-miR-148A-5p could inhibit cell proliferation by targeting Yes-associated protein 1 (YAP1) and PDPK1, respectively (62–64). Several viruses were found to encode miRNAs, targeting cellular or viral mRNAs to promote an intracellular environment favorable to the completion of the viral life cycle (65). Although virus-encoded miRNAs are rarely found in RNA viruses, a novel miRNA designated E (XSR) miRNA was identified in ALV-J-transformed turkey macrophage cell lines IAH30 (66). This miRNA play a critical role in the carcinogenicity of certain chicken genetic lines (67). More effort will be required to screen ALV-encoded miRNAs and analyze their functions during ALV infection.

LncRNA has been confirmed to regulate viral replication. Lnc-LTR5b derived from endogenous retrovirus LTR is located in the cytoplasm and competitively binds to the binding immunoglobulin protein (BiP), which is the main regulator of endoplasmic reticulum (ER). lnc-LTR5B serves as a competing endogenous RNA for BiP, restricting its physical availability. Upon ALV-J infection, the expression of lnc-LTR5b was inhibited, released BiP, and ftranslocated to the cell surface, facilitating viral entry (68). CircRNAs usually act as the sponge of microRNA (miRNA) in cancer. Our previous study reported that circ-Vav3 acts as a sponge for gga-miR-375. Upon ALV-J infection, gga-miR-375 was significantly down-regulated, while circ-Vav3 was up-regulated. The circ-Vav3/gga-miR-375 and its target YAP1 induces epithelial-mesenchymal transition (EMT) through influencing EMT markers to promote tumorigenesis (62, 63). The non-coding RNAs play multiple roles in ALV replication. However, how ALV regulates these non-coding RNAs to be beneficial or harmful RNAs needs to be clarified.

### Cellular Proteins and Signaling Pathways Associated With Viral Replication

Viruses could exploit the host cellular machinery for their replication. There are increasing evidence for the cellular signal transduction pathway involving viral replication. The phosphatidylinositol 3-kinase/serine-threonine protein kinase (PI3K/Akt) pathway was activated in cells infected with ALV-A, ALV-B or ALV-J, the activation of the PI3K/Akt signaling pathway is vital for viral entry (69). Wnt/β-catenin signaling pathway is a highly conserved pathway related to a variety of biological processes, the activation of this pathway benefits ALV-J replication. Viral titers were decreased when the Wnt/β-catenin signaling pathway was inhibited (70). The chicken telomerase reverse transcriptase (chTERT) might play a regulatory role in the process. chTERT is mutually regulated with the Wnt/β-catenin signaling pathway to inhibit apoptosis, promote ALV-J replication, and increase telomerase activity (71). The activation of the ERK/MAPK pathway is required for ALV-J replication and is associated with virus-induced tumorigenesis (72). Similarly, inhibition of the ERK/MAPK pathway suppressed ALV-A and ALV-B replication (73). Additionally, viral protein gp85 and p27 increase the production of IL-6 through activating the NF-κB/PI3K pathway and then induces the expression of vascular endothelial growth factor (VEGF)-A and its receptor VEGFR-2 in vascular endothelial cells and embryonic vascular tissue, promoting tumorigenesis (74).

In addition to the signalling pathway, several host proteins have been confirmed to inhibit viral replication. The oncogene p53 transcription factor recruits histone deacetylase 1 and 2 (HDAC1/2) to shut off the promoter activity of ALV integration region (75). Chicken tripartite motif-containing 62 (TRIM62) inhibits ALV-J replication through the SPRY structural domain (76), and chicken TRIM25, a member of the same tripartite motif (TRIM) family, also inhibits ALV-A replication by regulating MDA5 -mediated type I IFN response (77). In addition, ALV also utilize host proteins to promote its replication. Disruptor of telomeric silencing 1-like (DOT1L) was up-regulated during ALV-J infection in chicken macrophage HD11 cells, inhibition of DOT1L activity or deletion of DOT1L significantly reduced ALV-J replication by inducing the expression of IFN-β and ISGs (78). Cytokine signal-transduction inhibitor molecule 3 (SOCS3) promotes the replication of ALV-J by inhibiting the phosphorylation of JAK2/Stat3 (79). Similarly, cytokine-inducible srchomology2 (SH2)-containing protein (CIS) inhibits cytokine signaling, enhancing ALV-J replication (80). The collagen triple helix repeat containing-1 (CTHRC1) was moved from the nucleus to the cytoplasm to bind to SU, promoting viral replication (81, 82). Doublecortin-like kinase 1 (DCLK1) can interact with ALV-J SU, accelerating cellular progression from G0/G1 to the S phase, promoting cell proliferation. Moreover, the interaction increased the expression and accumulation of DCLK1, promoting epithelial-mesenchymal transition (EMT) by increasing N-cadherin, vimentin, MMP2, and transcription factor Snail1 and decreasing the expression of epithelial marker E-cadherin (83).

During ALV-J infection, apoptosis was associated with miRNAs and the GADD45β/MEKK4/p38MAPK signaling pathway. Over-expression of gga-miR-221 and gga-miR-222 promotes the proliferation, migration and growth of DF-1 cells, and decreased the expression of BCL-2 modifying factor (BMF), causing the strong anti-apoptotic ability (84). In ALV-J infected cells, the expression of miR-125b was down-regulated and its target Semaphorin-4D (Sema4D) was increased (85). When miR-125 decreased or Sema4D increased in HP45 cells, apoptosis was inhibited. These findings provide clues that ALV-J inhibits apoptosis by reducing the expression of miR-125. GADD45β was identified as a resistance factor involved in host resistance to ALV-J. When cells are infected with ALV-J, GADD45β binds to MEKK4, then inhibits autophagy and subsequently induces apoptosis (86).

## Future Perspectives

To data, there have been no vaccines or drugs available for the control of this disease. The most effective way is the differentiation and eradication of ALV-infected individuals in the population. Development of vaccines and screening effective drugs against ALV is of benefit to control this disease in the future. As an avian retrovirus, ALV integrates its genome into the host genome, causing vertical transmission in chickens. In order to complete viral replication and transmission, ALV must utilize a large arsenal of host factors. Studies on the interaction of ALV-host is benefit reveal the association of host factors with viral replication. ALV can adopt multiple proteins and non-coding RNA to accomplish viral replication. However, how does ALV inhibit host immune response and induce tumorigenesis? Why do the vaccines and drugs against ALV ineffective? Significant gaps still exist in understanding the underlying mechanism. More efforts will be required to identify more host factors associated with ALV replication and elucidate the effects of these factors on viral replication. Further findings of interactions between ALV and its cellular targets will benefit understanding the viral life cycle and the development of effective vaccines or drugs.

## Author Contributions

ST and WL conceptualized the mini-review topic. ST, JL, and WL contributed to writing the review. Y-FC and WL contributed to editing the review. All authors contributed to the article and approved the submitted version.

## Funding

This work was supported by the National Natural Science Foundation of China (32072842, 31802206), the Natural Science Foundation of Guangdong Province (2021A1515011147), and the Natural Science Foundation of Guangzhou (202102020942).

## Conflict of Interest

The authors declare that the research was conducted in the absence of any commercial or financial relationships that could be construed as a potential conflict of interest.

## Publisher’s Note

All claims expressed in this article are solely those of the authors and do not necessarily represent those of their affiliated organizations, or those of the publisher, the editors and the reviewers. Any product that may be evaluated in this article, or claim that may be made by its manufacturer, is not guaranteed or endorsed by the publisher.
